# Linear anti-jamming algorithm design for multi-channel optical fiber communication

**DOI:** 10.1371/journal.pone.0344533

**Published:** 2026-03-09

**Authors:** Haichao Yu

**Affiliations:** School of Computer and Information Engineering, Wuhan Railway Vocational College of Technology, Hubei, China; Beijing Institute of Technology, CHINA

## Abstract

The multi-channel fiber optic communication network, crucial for long-distance digital signal transmission, faces linear interference from orthogonal frequency division multiplexing. To address the challenge of linear anti-interference in digital signal transmission, this paper integrates Discrete Fourier Transform and Wavelet Transform techniques to precisely identify and locate linear interference signals during the transmission process of Orthogonal Frequency Division Multiplexing systems, and then specifically suppress and mitigate them. Firstly, this paper transforms multiple subcarrier digital signals in multi-channel transmission into linear interference signals. For these interference signals, wavelet decomposition is performed using wavelet transform technology, decomposing the noise signals into coefficients at different levels. Wavelet coefficients that are either above or below the threshold values are sequentially subjected to thresholding processing, thereby achieving denoising. Finally, by combining DFT and WT, the DFT-WT-LAJ algorithm is proposed. This algorithm searches for linear interference signals by employing both DFT and DMFT algorithms, and filters out all reconstructed linear interference signals from the transmitted signals. Experimental data show that the algorithm controls the amplitude below 10 Hz, achieves a synchronization probability of 0.7 at a signal-to-noise ratio (SNR) of 35 dB, and maintains an interference-to-signal ratio above 31 dB, significantly enhancing signal quality and transmission reliability.

## 1. Introduction

Amidst the contemporary wave of scientific and technological advancements, multi-channel fiber optic communication networks find widespread application across cutting-edge domains such as IoT applications, smart city infrastructures, and machine communication [1]. Serving as the backbone of communication, these networks efficiently convey digital signals through optical fibers, facilitating rapid information interchange. Nonetheless, fiber-optic communication signals frequently exhibit notable fluctuations during transmission, thereby compromising communication stability and reliability to some extent [2]. Particularly in long-distance communication scenarios, fiber-optic communication networks encounter unprecedented challenges stemming from diverse intrusion modes and continually evolving intrusion methods. Compounded by recurrent network equipment failures, human errors in processing, and other uncontrollable variables, this scenario significantly impacts signal stability, leading to the frequent occurrence of anomalous data.

Addressing this issue, research scholars undertake comprehensive investigations from the vantage point of designing anti-interference algorithms for digital signal transmission [3]. By optimizing algorithms, they aim to enhance the anti-interference capability of optical fiber communication signals during transmission, reduce signal fluctuations, and ensure the stability and reliability of communication. However, most existing solutions, such as FFT-assWP [4], LSID [5], and IFNC-ekf [6], while addressing linear interference issues to a certain extent, still suffer from limitations including high computational complexity, imprecise interference detection, and the need to improve network transmission efficiency. Specifically, the FFT-assWP method employs a joint adaptive spectrum sensing technology that combines multi-threshold Fast Fourier Transform and wavelet packet. Although it can relatively accurately locate severely interfered frequency bands by setting high and low threshold values, it may fail to capture all interference signals, particularly those with relatively low interference-to-noise ratios. The LSID method proposes a low-complexity linear scanning interference detection approach that takes into account the probabilities of false alarms and missed detections; however, its process is overly cumbersome, which is not conducive to practical applications. The IFNC-ekf method, on the other hand, utilizes an iterative phase noise compensation method based on an Extended Kalman Filter. While it effectively suppresses the loss of received signal-to-noise ratio, the stability of its network transmission signal fluctuation amplitude remains to be verified.

Drawing from current research, it is established that the utilization of orthogonal frequency-division multiplexing (OFDM) in multichannel fiber optic communication networks characterizes it as an OFDM system [7]. The inherent advantages of the OFDM system, including ease of operation, compatibility with various channel transmission types, and robust resistance to multipath fading, have propelled it to become the predominant technology for transmission within multichannel fiber optic communication networks. Nonetheless, the occurrence of significant linear interference during OFDM transmission adversely affects its performance, prompting intensive investigation into linear suppression techniques and signal anomaly detection methods within related fields. Existing approaches predominantly leverage orthogonal frequency division multiplexing to obviate the need for high-pass filters for suppressing nearby interference and enhancing sensor dynamic range. Adjustments to the high peak-to-average power ratio are made to ameliorate signal quality through distance-frequency coupling [8]. However, these methodologies exhibit computational inefficiencies in handling linear interference. Notably, for non-stationary interference scenarios like linear modulation, Fourier Transform (FT) emerges as a promising avenue due to its capability to address diverse filtering complexities and optimize algorithmic processes [9]. Additionally, it is observed that extant studies overlook the susceptibility of fiber-optic communication signals to noise interference, such as electromagnetic radiation, prior to anomalous data detection in long-distance multichannel fiber-optic communication networks [10].

Therefore, in order to solve the problem of noise interference such as linear interference and electromagnetic radiation, this paper combined with discrete Fourier transform to efficiently detect linear interference signals, accurately search for linear interference signals in OFDM transmission of multi-channel optical fiber communication networks, and construct a fiber communication signal denoising method based on wavelet transform. A linear anti-jamming algorithm for digital signal transmission based on discrete Fourier transform and wavelet transform is implemented, namely, DFT-WT-LAJ. The paper offers the following specific contributions:

- Introduction a novel linear interference signal conversion algorithm tailored for multi-channel fiber optic communication network transmission. This paper employs the DFT to efficiently detect linear interference signals. It transforms the subcarrier digital signals in multi-channel transmission OFDM into linear interference signals, enabling precise searching for interference signals. This method overcomes the shortcomings of the traditional FFT method in capturing interference signals with low interference-to-noise ratios.
- Proposes a denoising technique for fiber optic communication signals grounded on wavelet transform. We constructs a wavelet transform-based denoising method for optical fiber communication signals. Through wavelet decomposition, the noise signals are broken down into coefficients at different levels, and thresholding processing is applied to wavelet coefficients that are either above or below the threshold values, thereby achieving signal denoising.
- Develops a linear interference signal detection algorithm hinging on discrete Fourier transform and two-step discrete matched Fourier transform algorithms. This algorithm accurately identifies the presence of linear interference signals in the transmission OFDM of multi-channel fiber optic communication networks, reconstructs said signals, and subsequently filters out the reconstructed linear interference signals within the transmitted signals, thereby achieving linear interference suppression. This method not only achieves linear anti-interference without compromising useful signals, but also significantly reduces processing complexity and enhances the accuracy and efficiency of interference suppression.

## 2. Related works

The fiber optic channel boasts a substantial transmission capacity and reliable operation, offering unparalleled advantages compared to conventional communication methods. However, during transmission, optical fibers are susceptible to stronger interference from environmental factors and external influences. This susceptibility is particularly pronounced in the context of multi-channel fiber optic communication networks, which often exhibit orthogonal frequency division multiplexing characteristics. Consequently, in long-distance transmission scenarios, encountering significant linear interference within the OFDM system is inevitable.

To address the challenge of linear interference in OFDM systems, existing literature [4] proposed the employment of the multi-threshold Fast Fourier transform (FFT) [11] in conjunction with the wavelet packet joint adaptive spectrum sensing technique [12]. This approach facilitated accurate spectrum sensing, enabling the identification and elimination of linear interference to uphold communication stability and quality. Despite the effectiveness of FFT in pinpointing frequency bands with pronounced interference via dual threshold settings, it may fail to detect all interfering signals, especially those with lower signal-to-noise ratios. In response, additional literature [13] integrates wavelet packet spectral search techniques [14] to identify residual interference. Leveraging the multi-resolution property of wavelet packet analysis, this technique offers refined spectral insights across diverse frequency bands, enhancing the precision of interference detection.

In contrast, literature [5] advocated for the reorganization of the OFDM system based on search outcomes. By adjusting subcarrier configuration and power allocation to circumvent overlap with interference signal bands, this approach effectively sidesteps interference, dynamically adapting to evolving network environments to fortify communication system robustness. However, despite its merits, the precision of secondary linear interference searches remains insufficient. Given the intricate and variable nature of interference signals, certain interferences may still evade detection, consequently impeding the efficacy of linear interference suppression and compromising communication system performance.

In contrast, literature [15] introduced a low-complexity linear scanning interference detection method, LSID, tailored for addressing linear interference in multichannel communication. By accounting for the probabilities of false alarms and missed detections, LSID effectively mitigates linear interference, ensuring seamless information transmission. However, the method’s cumbersome process impedes its practical applicability in combating linear interference within multi-channel communication network transmission.

Furthermore, literature [16] delved into the intricacies of cochannel interference, deriving the signal-to-dry ratio expression for echo signals through rigorous signal-to-dry ratio analysis. Building upon this foundation, literature [17] innovatively devised a cochannel interference suppression algorithm by amalgamating interleaved multiple access concepts with interference reconstruction within the OFDM framework. Through adept utilization of diverse interleaving methods to differentiate node signals, this algorithm enhances signal recognizability and anti-interference capabilities. Moreover, by introducing an interference cancellation algorithm predicated on interference reconstruction [18], the scheme accurately identifies and eliminates interference signals, effectively suppressing cochannel interference. However, despite its theoretical advancements, the algorithm’s network transmission efficiency warrants further enhancement to optimize practical application outcomes.

In an effort to address these challenges and enhance the precision of interference reconstruction while exploring more efficient interference cancellation strategies, literature [21] introduced an iterative phase noise compensation method [22] employing an extended Kalman filter to calculate the initial value of the orthogonal frequency division multiplexing radio backhaul link. This approach iteratively updated the time-varying phase noise estimate and variance in the time domain, thereby reducing computational complexity and effectively suppressing loss in received signal-to-noise ratio. However, verification regarding the stabilization of signal fluctuation amplitudes transmitted by the network remains pending.

Moreover, within communication systems, signal transmission processes are often susceptible to various forms of linear interference stemming from channel instability, equipment malfunctions, or external sources [20]. Traditional anti-interference methodologies typically entail a trade-off between signal processing complexity and interference suppression efficacy. For instance, literature [19] leveraged orthogonal frequency division multiplexing to obviate the need for high-pass filters for close target suppression, thereby enhancing sensor dynamic range. While adjusting the high peak-to-average power ratio improves signal quality via distance-frequency coupling, it engenders computational arithmetic challenges.

Table 1 summarizes key characteristics, computational complexities, and performance metrics of various linear interference suppression methods in multi-channel optical fiber communication networks. Notably, while traditional methods like FFT-assWP and high-pass filters suffer from high computational complexity or imprecise interference detection, the proposed DFT-WT-LAJ algorithm stands out. It optimizes computational load while significantly enhancing signal quality and synchronization probability, addressing the critical challenge of linear interference in optical fiber communications. In order to balance the problem of anti-jamming capability and computational arithmetic, we have thoroughly studied and found that the construction of linear anti-jamming algorithms based on the Fourier algorithm has significant advantages. As a powerful mathematical tool, the Fourier algorithm is able to discretize finite-length non-periodic signals in both time and frequency domain dimensions, which provides the feasibility of signal analysis by computer. Through the Fourier transform, we can convert a continuous time signal into a continuous spectrum, and then analyze the characteristics of the signal in the frequency domain. At the same time, because the Fourier algorithm can discretize the signal, which makes the computer can efficiently deal with large-scale signal data, improving the computational power. More importantly, the linear anti-interference algorithm constructed based on Fourier algorithm is capable of searching out the linear interferences generated by the discretization of the continuous time signal and the continuous frequency spectrum. Therefore, this paper will control the linear interference within a certain range by accurately identifying these interference components and taking targeted measures for interference suppression.

**Table 1:** Comparison of related studies.

Method Name	Characteristics	Computational Complexity	Performance Metrics
FFT-assWP [4]	Combines FFT with wavelet packet for spectrum sensing	High	Locates severely interfered frequency bands, misses low SNR interference
LSID [5]	Low-complexity linear scanning interference detection	Low	Addresses linear interference, imprecise in secondary search
IFNC-ekf [6]	Iterative phase noise compensation	High	Suppresses SNR loss, signal fluctuation to be verified
Traditional High-Pass Filter Methodp [19]	Uses high-pass filters to suppress interference	Moderate	Enhances dynamic range, poor computational efficiency
Co-channel Interference Suppression Algorithm [20]	Interleaved multiple access with interference reconstruction	High	Improves signal identifiability, transmission efficiency to be enhanced
DFT-WT-LAJ (Ours)	Combines DFT with wavelet transform for anti-jamming	Optimized and reduced	Enhances signal quality, reduces bit error rate, high synchronization probability

## 3. Model design

**Fig 1:**
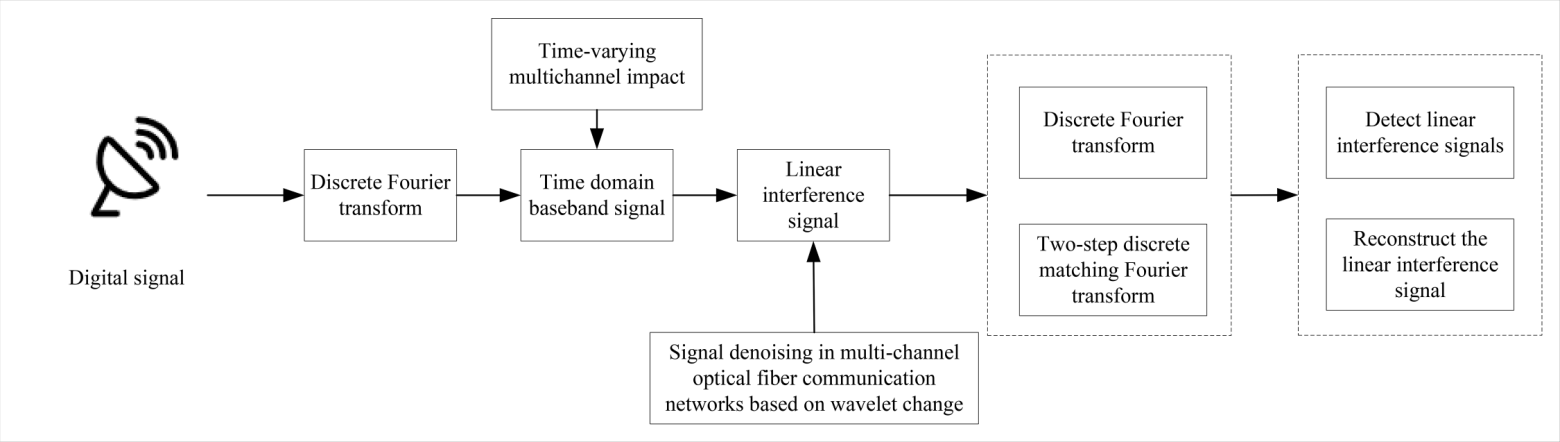
Model framework.

The transmission process of a multi-channel fiber optic communication network constitutes an OFDM system. Addressing the challenge of suppressing linear interference inherent in transmission necessitates the initial conversion of the linear interference present in multi-channel fiber optic communication network transmission signals. The digital signal linear anti-interference algorithm DFT-WT-LAJ, as devised in this paper and depicted in Fig 1, encompasses several key steps. Firstly, the discrete Fourier transform is employed to derive the time-domain baseband signal, which is then combined with the time-varying impacts across multiple channels to ascertain the linear interference signal. Subsequently, a denoising method for the linear interference signal is formulated based on wavelet variation, enhancing the independence across all decomposition scales of the fiber optic communication signal. Finally, leveraging the denoised linear signals, the discrete Fourier transform and two-step discrete matched Fourier transform algorithms are synergistically applied to efficiently detect and reconstruct the linear interference signals, thereby achieving the objective of linear interference suppression.

### 3.1. Transmission of linear interference signal conversion

For the transmission of digital signals in a multichannel environment with OFDM subcarrier number M, let the transmitted modulation symbol be denoted as H. The application of discrete Fourier transform results in the time domain baseband signal as:


$$\begin{array}{c} S(t)=\frac{\text{1}}{\sqrt{M}}\sum_{n=\text{1}}^{\infty }e^{j*\text{2}\pi }H_{k}(t-n*f_{k}) \end{array}$$ (1)

Where j denotes the imaginary number, t denotes the sampling time interval, and n denotes the discrete signal period. $f_{k}$ denotes the center frequency of the kth subcarrier. To remove the interference from the presence of symbols, it is necessary to set the cyclic prefix to $M_{k}$ sampling points at the end of the time domain baseband signal. Let $L_{t}$ denote the number of multi-channels, $\varphi_{p}$ denote the fading factor in the pth channel, u denotes the Doppler shift, and $t_{p}$ denotes the time delay denotes the fading factor in the household channel. Then the impulse response of the time-varying multichannel is:


$$\begin{array}{c} (t)=\sum_{p=\text{1}}^{L_{t}}\varphi_{p}e^{j*\text{2}\pi }u(t-t_{p}) \end{array}$$ (2)

Then, the baseband transmission signal in the receiver is obtained by removing the cyclic prefix:


$$\begin{array}{c} y(t)=\sum_{p=\text{1}}^{L_{t}}\varphi_{p}e^{j*\text{2}\pi *\Delta f}*u(t-t_{p})*\alpha \end{array}$$ (3)

Where Δf denotes the frequency deviation between the receiver and transmitter carrier oscillators, and  α denotes the additive Gaussian white noise. The discrete Fourier transform method is applied to start sampling y(t), then the linear interference signal D is:


$$\begin{array}{c} D=\sum_{p=\text{1}}^{L_{t}}\varphi_{p}e^{j*\text{2}\pi *\Delta f*\frac{\beta *\chi }{\lambda *M}}*u[k-k_{p}]*\alpha \end{array}$$ (4)

Where β denotes the normalized Doppler shift under the p-th path,  χ denotes the normalized carrier frequency deviation, and  λ denotes the subcarrier spacing.

### 3.2. Wavelet transform based denoising for multi-channel fiber optic communication signals

Within the multichannel fiber optic communication signal, the noise signal undergoes wavelet decomposition, resulting in the extraction of difference level coefficients. Subsequently, wavelet coefficients exceeding or falling below the predefined threshold are sequentially subjected to thresholding. The resultant fiber optic communication signal undergoes reconstruction to restore the original signal, thereby accomplishing signal denoising. Let the noise signal r(y) be present within the linear interference signal D:


r(y)=g(y)+ε*d(y),y=1,2,...,M−1 (5)

Where the communication signal of a multi-channel fiber optic communication network is  g(y), and the Gaussian white noise and noise signals in the data of the fiber optic communication network are in order  d(y),r(y). In the algorithm proposed in this paper, the selected wavelet type is the Daubechies wavelet. The Daubechies wavelet possesses excellent properties such as compact support and orthogonality, which enable it to strike a good balance between time-frequency localization capabilities. It excels in signal denoising and feature extraction, making it well-suited for addressing linear interference issues in optical fiber communication signals and effectively enhancing signal quality. The sampling point of the fiber optic communication signal with consistent spacing is y, and  ε is the noise level. If the discrete sampling data of the noise signal  r(y) is $\;r_{k}$. Assuming that $\omega^{\text{1}},\omega^{\text{2}}$ are the scale factor, wavelet coefficient in order. $q_{m-\text{2}z}$,${\;f}_{m-\text{2}z}$ is the orthogonal mirror filter bank. n and m are the number of wavelet decomposition layers and the number of discrete sampling points of the noise signal in the optical fiber communication signal in order. $w_{n,z}^{\text{1}}$, $w_{n,z}^{\text{2}}$ are the scale coefficients of the mth signal discrete sampling point of the n-1-th layer, and the wavelet coefficients of the mth signal discrete sampling point of the n-1st layer, in that order. Then the decomposition method of orthogonal wavelet transform of noise signal r(y) is shown in Formular (6) and (7):


$$\begin{array}{c} w_{n,z}^{\text{1}}=\sum_{m=-\infty }^{\infty }w_{n-\text{1},m}^{\text{1}}q_{m-\text{2}z} \end{array}$$ (6)

$$\begin{array}{c} w_{n,z}^{\text{2}}=\sum_{m=-\infty }^{\infty }w_{n-\text{1},m}^{\text{2}}f_{m-\text{2}z} \end{array}$$ (7)

Subsequently, orthogonal wavelet decomposition is performed on the fiber optic communication signal, employing both low-pass and high-pass filters for filtering. The filtered output yields the low-frequency profile and high-frequency details of the long-distance multichannel fiber optic communication signal. With each orthogonal wavelet decomposition, the implementation of decomposition is no longer required for the high-frequency portion, as it is based on the low-frequency portion of the main part. The multichannel fiber optic communication signal is then reconstructed as:


$$\begin{array}{c} w_{n,z}^{\text{1}}=\sum_{m=-\infty }^{\infty }\left(w_{n,m}^{\text{1}}q_{m-\text{2}z}+w_{n,m}^{\text{2}}f_{m-\text{2}z}\right) \end{array}$$ (8)

Following the decomposition of the noisy signal into various frequency bands at a particular scale, the frequency band containing the noisy signal is nullified and subsequently reconstructed. This process facilitates the denoising of optical fiber communication signals.

### 3.3. Design of DFT-WT-LAJ algorithm

In the subsequent sections, we formulate the DFT-WT-LAJ algorithm by utilizing the denoised signal as established in Sections 3.1 and 3.2. Given that linear interference signals with constant amplitude manifest continuous band characteristics in the frequency domain, we consider the two extreme values of the aforementioned linear interference signal, i.e., $f_{max}$ and $f_{min}$, can be derived by changing the sampling frequency and the duration of the linear interference signal. The broadband of the linear interference signal is calculated from the extreme values:


$$O=f_{max}-f_{min}$$ (9)

Next, the frequency of modulation of the linear interference signal is derived using the discrete short-time Fourier transform:


$$h_{\text{1}}=\frac{O}{T_{Ls}}$$ (10)

Where $T_{Ls}$ represents the length of the time period of the sampled linear interference signal. $h_{\text{1}}$ represents a coarse estimate of the tuning frequency of the linear interference signal. After the coarse estimation. After searching for the tuning frequency in a small range centered on $h_{\text{1}}$, the discrete matched Fourier transform method is applied to make a fine estimation of the linear interference signal. At this time by discrete two-step matched Fourier transform can be rewritten as:


$$\begin{array}{c} W(f,h)=\int_{\text{0}}^{T}s(t)e^{-j*\text{2}\pi *f}dt \end{array}$$ (11)

Where s(t) is calculated as follows:


$$s(t)=w(t)e^{-j*\text{2}\pi *(\frac{\text{1}}{\text{2}h})*t}$$ (12)

**Fig 2:**
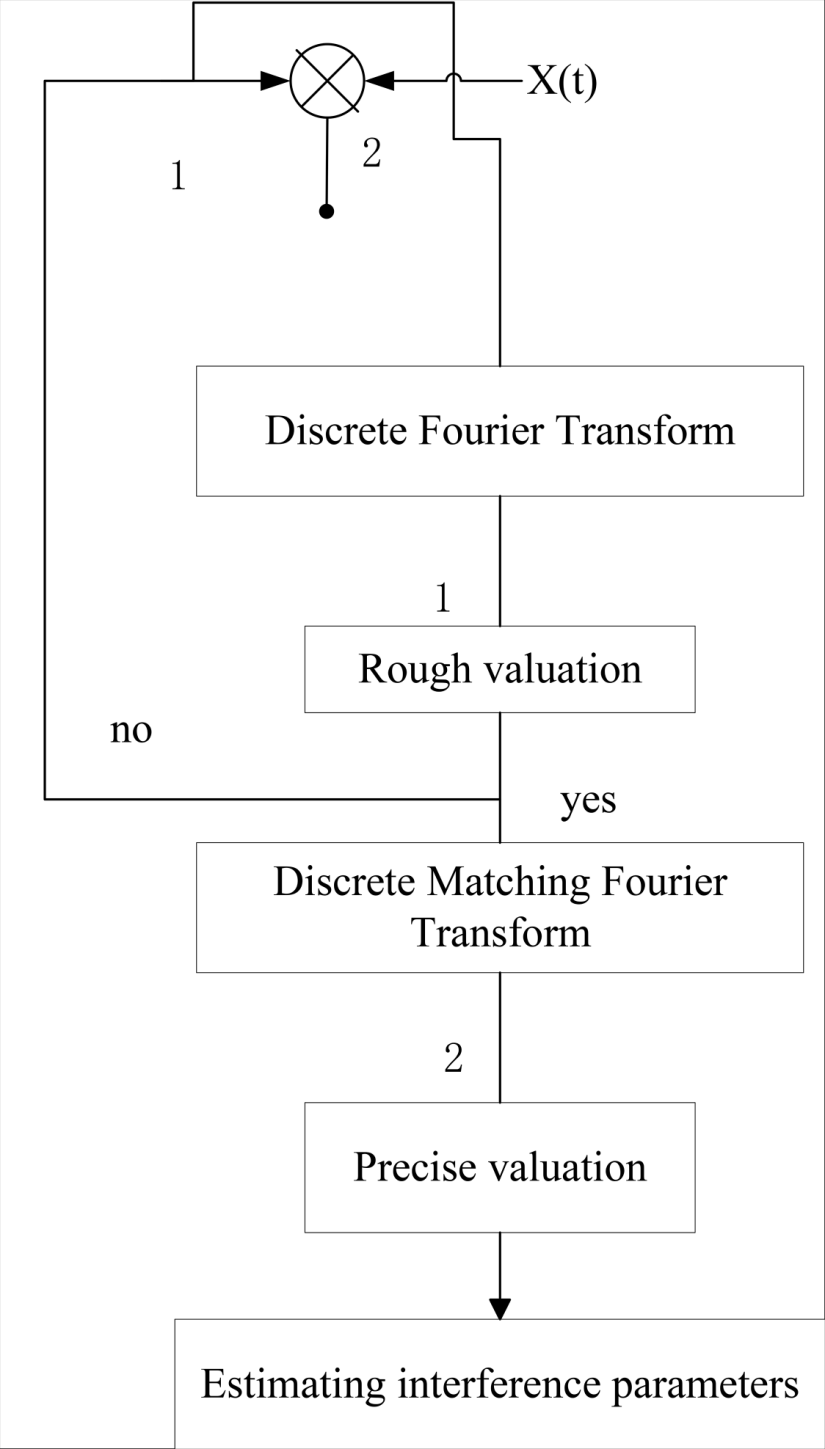
Linear interference signal estimation based on Fourier algorithm.

The two-step discrete matching Fourier transform first roughly estimates the linear interference modulation frequency through DFT, then conducts a precise search in a small range, and combines the matching Fourier transform to obtain the maximum magnitude value, achieving precise estimation. This method is more accurate than the traditional DFT method. In the multichannel transmission OFDM context, the discrete Fourier transform and discrete matched Fourier transform algorithms are employed to undertake the specific estimation process of linear interference signals, as depicted in Fig 2. Initially, the signal undergoes transformation via discrete Fourier transform to obtain a preliminary estimation of the tuning frequency of the linear interference. Next, the FM range is set at $\left[h_{\text{1}}-\varepsilon ,h_{\text{1}}+\varepsilon \right]$. Set ${\;h}_{\text{1}}-\varepsilon$ as the starting point, and step by step with u. When the operation reaches the k-th time, the tuning frequency of s(t) in Eq. $h_{\text{1}}+\varepsilon -k_{u}$ is obtained. Formula (12) is used to compute s(t). Through discrete Fourier transform, we obtain the maximum amplitude of the transformed spectrum in this operation, denoted as R. Simultaneously, the frequency estimation value at the frequency point is recorded. This process is repeated iteratively until the frequency search is completed, comparing all R values. The maximum value is identified, and the corresponding frequency value is utilized as the initial frequency. Subsequently, discrete matched Fourier transform is employed for precise estimation, enabling the identification of all linear interference signals present in the multichannel fiber optic communication network transmission process.

**Fig 3:**
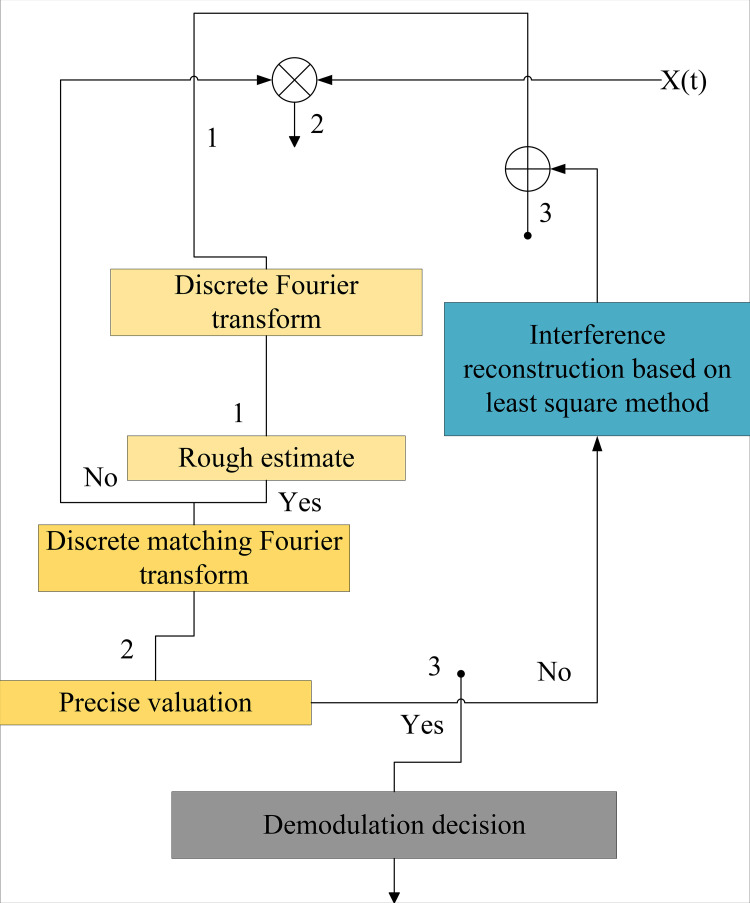
Linear interference suppression.

To effectively mitigate linear signal interference, it is imperative to meticulously filter out all reconstructed linear interference components from the transmitted signal. To achieve this objective, we have devised a meticulously crafted linear interference suppression algorithm, as illustrated in Fig 3. At the heart of this algorithm lies the precise estimation of signal amplitude parameters, accomplished through subtle transformations of the linear interference signal using the least squares method. Once these critical amplitude parameters are determined, the linear interference signal can be accurately reconstructed based on them. Subsequently, these reconstructed linear interference signals are precisely filtered out from the transmitted signals, thereby successfully suppressing linear interference signals. This process not only enhances the quality of signal transmission but also ensures the accuracy and stability of communication.

## 4. Experiments and analysis

In this section, we conduct a comprehensive comparison and analysis of the proposed DFT-WT-LAJ algorithm. The analysis primarily focuses on evaluating the algorithm’s denoising performance during digital signal transmission anti-jamming, linear interference signal detection, and linear interference signal reconstruction. We compare the performance of our algorithm with existing methods such as FFT-assWP, 9 LSID, 15 and IFNC-ekf19 under simulated experimental conditions. Various performance indicators are examined to assess the efficacy of each algorithm. Finally, we assess the signal denoising performance to validate the feasibility of the wavelet transform-based multichannel fiber optic communication signal denoising method proposed in this paper.

### 4.1. Experimental setup

We conducted simulation tests using the Madab2019 toolbox, sampling a multi-channel fiber-optic communication network OFDM system with uniform parameters across all channels. The channel sampling frequency was set to 15 MHz, the number of OFDM subcarriers to 2048, and the sequence length to 203. The experiments focused on assessing linear interference present in the channel within this environment.

In constructing the experimental setup, we realized the multi-channel fiber optic communication network equipment through fiber optic connections and linear anti-interference device connections. We adjusted the signal generator to generate the data signal to be transmitted, introducing signal interference at different frequencies. The signal was then input into the linear anti-interference device for conversion. Subsequently, the converted anti-jamming signal was transmitted through the multi-channel fiber-optic communication network equipment. The optical power of the signal was detected using an optical power meter, and the spectrum of the transmitted signal was analyzed using a spectrum analyzer. Numerical results were recorded, and the experimental results presented below represent the average of the numerical results obtained after 100 runs.

### 4.2. Evaluation indicators

The synchronization probability serves as a pivotal metric for assessing the synchronization status among channels within a multichannel network transmission environment. In such systems, the synchronization status directly influences the transmission efficiency of the entire network. A higher synchronization probability indicates improved synchronization among channels, effectively ensuring the coherence and accuracy of data transmission. Consequently, the multichannel network can process and transmit data more efficiently, thereby enhancing the overall system transmission efficiency. Moreover, a higher synchronization probability signifies reduced interference on each channel, thereby bolstering the stability and reliability of data transmission. Generally, a synchronization probability exceeding 0.5 suggests a favorable transmission environment capable of supporting efficient and stable data transmission.

Conversely, the Jam-to-signal ratio is a crucial parameter for evaluating the quality of suppressed linear signals. In multichannel communication systems, linear interference poses a common threat to signal quality. By employing effective linear anti-interference methods, the impact of this interference can be mitigated, thereby enhancing signal quality. A low Jam-to-signal ratio for the suppressed linear signal (e.g., 25 dB or less) indicates significant success in mitigating the impact of linear interference on the signal. A lower Jam-to-signal ratio signifies higher signal purity and superior anti-linear interference effects, thereby ensuring the accuracy and reliability of data transmission.

### 4.3. Analysis of signal processing performance

**Fig 4:**
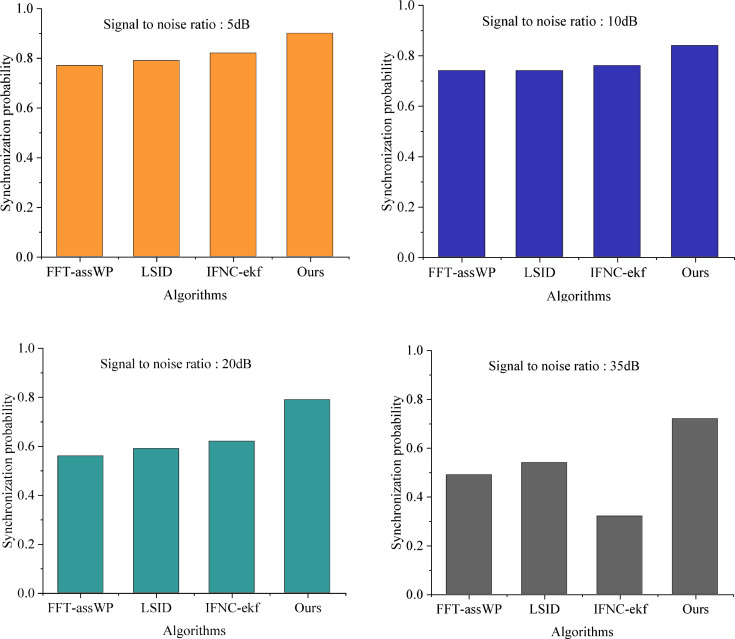
Comparison of network transmission efficiency among different algorithms.

In the network transmission efficiency comparison experiments conducted across different signal-to-noise ratio (SNR) environments, the results depicted in Fig 4 illustrate notable trends. As the SNR increases, the synchronization probability of all four methods exhibits a declining trend. Specifically, the FFT-assWP algorithm decreases to 0.49 when the SNR is 35 dB, and the IFNC-ekf algorithm decreases to 0.32 under the same SNR condition, failing to meet the synchronization probability criterion of 0.5 for both methods.

Conversely, at a SNR of 35 dB, the synchronization probability reaches 0.7, representing an approximately 43% improvement over FFT-assWP (0.49) and about a 35% improvement over LSID (0.52). The interference-to-signal ratio consistently remains above 31 dB, outperforming both LSID and IFNC-ekf. After linear interference suppression, the amplitude is controlled below 10 Hz, exhibiting greater stability compared to the maximum of 22 Hz achieved after IFNC-ekf processing. The performance gain is significant, highlighting the effectiveness of this algorithm.

**Fig 5:**
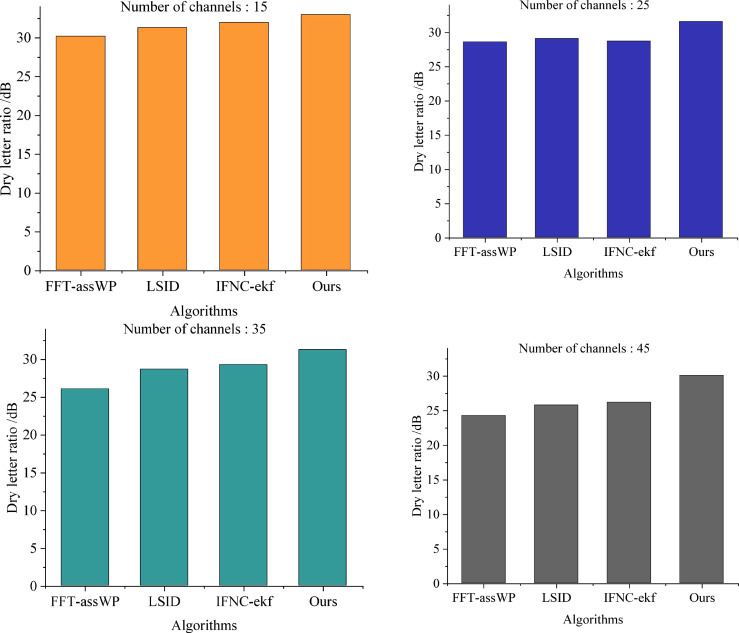
Comparison of linear anti-jamming performance among different algorithms under different number of channels.

We conducted experiments where we set the search radius to 20 meters to evaluate the efficacy of the four methods in suppressing the detected linear interference signal. The results are depicted in Fig 5.

From Fig 5, it is evident that there is a decreasing trend in the signal-to-jamming ratio (SJR) values of all four methods with the increase in the number of channels. Our proposed method effectively suppresses transmitted linear signals in the multichannel fiber optic communication network, consistently maintaining a high signal-to-jamming ratio (SJR) exceeding 30 dB. In contrast, although the LSID and IFNC-ekf algorithms achieve a SJR meeting the requirement of 25 dB, their SJR remains significantly lower than that of our proposed method.

This disparity can be attributed to the utilization of the least squares method in our proposed method to accurately calculate the amplitude of the linear interference signal, thus enabling effective suppression. These results demonstrate the efficacy of our proposed method in mitigating signal fluctuations in multichannel fiber optic communication network transmission and optimizing the network transmission environment.

### 4.4. Analysis of linear anti-jamming ability

Since the performance difference between the DFT-WT-LAJ algorithm and the IFNC-ekf algorithmin the analysis of Section 4.3 is not very apparent, further illustration of the algorithm’s advancement is warranted. We conducted additional experiments by adding linear interference signals to the established environment. The amplitude of the digital signal spectrum exhibits significant improvement with increasing normalized frequency after the addition of interference signals.

**Fig 6:**
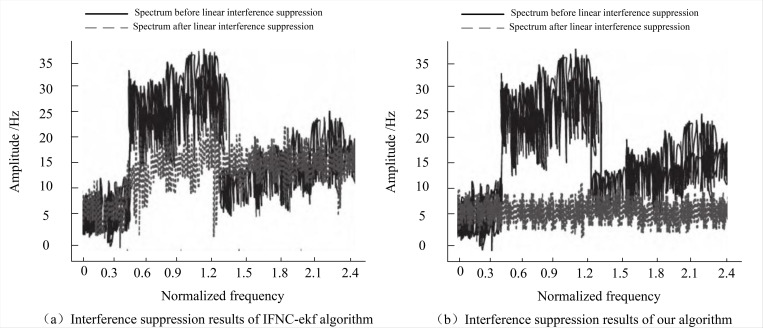
Linear anti-jamming ability among different algorithms.

By running the DFT-WT-LAJ algorithm and IFNC-ekf algorithm respectively, the results depicted in Fig 6 were obtained. It is evident that the DFT-WT-LAJ algorithm of this paper demonstrates smoother amplitude fluctuations, remaining controlled below 10 Hz after linear interference suppression for multichannel fiber optic network transmission, effectively suppressing amplitude interference. In the contrast, the amplitude fluctuations in the digital signal spectrum processed by the IFNC-ekf algorithm are notably more pronounced, reaching peak values of 20 Hz and 22 Hz. The signal undergoes more extensive modification, and stability is compromised at higher normalized frequencies, making it unreliable for practical applications.

The proposed DFT-WT-LAJ algorithm comprehensively utilizes the discrete Fourier transform method to convert the linear interference signal. It achieves coarse estimation through signal denoising and the calculation of its tuning frequency and combines with the discrete matched Fourier transform method to obtain the maximum magnitude of the transformed spectrum. This process facilitates fine estimation of the linear interference signal, enhancing the algorithm’s ability in linear anti-jamming ability.

### 4.5. Analysis of signal denoising performance

**Fig 7:**
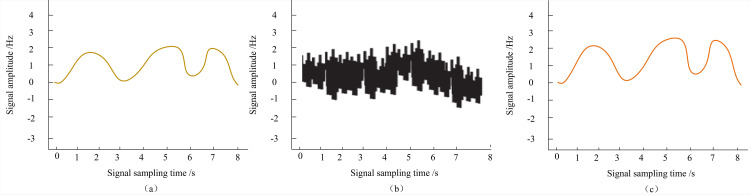
Comparison of (a) original signal, (b) noisy signal and (c) denoised signal.

To comprehensively assess the practical efficacy of the method proposed in this paper for denoising fiber optic communication signals, we present the original form of the fiber optic communication signal in sub-figure (a) of Fig 7. This signal appears clear and stable, characterized by distinct waveforms and features.

However, upon introducing linear interference into the system, the communication signal is significantly impacted, resulting in a noisy signal as depicted in sub-figure (b) of Fig 7. In this figure, it is evident that the original signal’s features are obscured by noise, rendering the signal waveform cluttered and challenging to discern.

Subsequently, we applied the denoising method proposed in this paper to process the fiber-optic communication signal disturbed by noise. The morphology of the processed signal is shown in sub-figure (c) of Fig 7. Upon close examination, it is evident that the denoised signal, processed using the method proposed in this paper, closely mirrors the original signal. The noise has been effectively suppressed and eradicated, reinstating the clarity and distinct recognizability of the signal’s waveform and features. The denoising performance is exceptionally satisfactory.

This outcome unequivocally demonstrates the superiority and effectiveness of the method proposed in this paper for denoising optical fiber communication signals in the presence of linear interference. Such efficacy provides robust support for enhancing the performance and quality of optical fiber communication networks.

## 5. Conclusion

Addressing the linear interference challenges posed by OFDM in multi-channel fiber optic communication networks, this paper introduces an innovative linear anti-interference method. By integrating the discrete Fourier transform with the discrete matched Fourier transform, we accurately identify and locate linear interference signals in OFDM system transmission processes, effectively suppressing and attenuating them. To further enhance signal transmission reliability and network performance, we incorporate a denoising step and utilize the least-squares method to accurately estimate the amplitude of interfering signals, subsequently filtering them out from the original transmitted signals. In comparison with traditional methods, this approach not only achieves linear anti-interference without compromising useful signals but also significantly reduces processing complexity. Demonstrating exceptional interference suppression capability, high transmission efficiency, superior dry signal-to-signal ratio performance, and notable denoising effects, this method holds promising prospects in multichannel fiber optic communication network transmission. From the perspectives of communication security and anti-interference capability, the algorithm proposed in this paper holds immense potential when deeply integrated with cutting-edge research. By introducing a probabilistic framework [22], its robustness against detection can be enhanced, providing a powerful complement to existing linear anti-interference frameworks. Additionally, the adoption of adaptive anti-interference techniques and learning-based interference mitigation strategies [23] can also offer new insights. In the future, integrating these ideas could pave the way for developing more secure, efficient, and highly resilient communication strategies.

## Supporting information

S1 Data Datalink. This is the datalink used in this work.
(TXT)

S2 Code Code. This is the code of the manuscript.
(RAR)
